# Community health and well-being workers (CHWWs) for preventive healthcare services uptake in the UK: a cluster-randomised controlled trial protocol

**DOI:** 10.1136/bmjopen-2026-124582

**Published:** 2026-10-23

**Authors:** Rawan Gadi, Cornelia Junghans Minton, Ahmad Alkhatib, Thomas Woodcock, David Peiris, Otavio Berwanger, Laurent Billot, Matthew Harris

**Affiliations:** 1 Department of Primary Care and Public Health, Imperial College London, London, UK; 2 Dental Public Health Department, King Abdulaziz University, Jeddah, Saudi Arabia; 3 University of West London, London, UK; 4 School of Public Health, Imperial College London, London, UK; 5 The George Institute for Global Health, Sydney, New South Wales, Australia; 6 Imperial College London, London, UK; 7 Faculty of Medicine and Health, The George Institute for Global Health, UNSW Sydney, New South Wales, Australia; 8 Statistics Division, The George Institute for Global Health, Newtown, New South Wales, Australia

**Keywords:** Primary Care, Health Services, Health Workforce, Preventive Health Services, Delivery of Health Care, Integrated, Protocols & guidelines

## Abstract

**Introduction:**

Recognised within the National Health Service (NHS) 10-year plan as an innovative workforce solution to enable the left shift care towards integrated, neighbourhood models, community health and well-being workers (CHWWs) are rapidly expanding across England. Inspired by Brazil’s Family Health Strategy, the model has scaled from an initial pilot in Westminster to over 28 localities. However, despite this national expansion, robust empirical evidence of its impact remains limited. This protocol describes a pragmatic cluster randomised controlled trial in Westminster designed to provide definitive evidence on whether embedding CHWWs within local communities effectively improves the uptake of preventive healthcare services.

**Methods and analysis:**

A pragmatic, parallel-group, superiority cluster-randomised controlled trial is being conducted using routinely collected electronic health records. 60 geographically defined ‘village’ clusters (approximately 120 households in each cluster, 7200 households in total) located in socioeconomically deprived areas of Westminster, London, were randomised (1:2 allocation ratio) to either the intervention or control group. The intervention involves locally recruited CHWWs conducting comprehensive, proactive monthly household visits to promote preventive care and address social determinants of health between January 2024 and December 2025. The control group receives usual care. The primary outcome is the Composite Referral Completion Indicator (CRCI), defined as the household-level rate of completed eligible preventive healthcare services per person-year (including immunisations, cancer screenings and NHS Health Checks). Secondary outcomes include individual preventive service uptake and healthcare utilisation metrics (eg, general practice and accident and emergency attendances). Outcomes will be extracted from the Whole Systems Integrated Care (WSIC) database. Primary analysis will follow the intention-to-treat principle using generalised linear mixed models, supplemented by hierarchical targeted maximum likelihood estimation for absolute effects.

**Ethics and dissemination:**

The study relies on de-identified, routinely collected data to evaluate a new routine primary care service. Individual informed consent was waived because the use of retrospective routinely collected data for analysis fell under the existing global ethics agreement for the WSIC database (REC reference: REC18/WM/0323/IRAS252449). Nevertheless, the study was reviewed and approved by the Health Research Authority (HRA 23/HRA/4972; 20 December 2023). Results will be disseminated through peer-reviewed academic publications, conference presentations and plain-language summaries shared with the local community.

**Trial registration number:**

NCT06144580.

**STRENGTHS AND LIMITATIONS OF THIS STUDY**

- The pragmatic, cluster-randomised design conducted within routine primary care settings strongly improves the external validity of the findings.
- Using the comprehensive Whole Systems Integrated Care database to establish a fixed point-in-time cohort allows the study to capture data on all eligible residents without requiring individual consent, thereby eliminating selection and survivorship biases.
- The use of the Composite Referral Completion Indicator is a major strength as it effectively captures the holistic, multidomain impact of the community health and well-being workers (CHWWs) on preventive care, though this relies heavily on the administrative accuracy and completeness of the routine electronic health records.
- While randomising at the village/cluster level was specifically chosen to minimise contamination between study arms, there remains an inherent risk of social contamination; the community-based nature of the intervention makes it easy for engaged residents to share health advice and influence the preventive health behaviours of relatives, friends or neighbours residing in nearby control clusters.
- The study is conducted within the London Borough of Westminster, targeting the most socioeconomically deprived lower super output areas; while this focus strengthens internal validity and relevance to deprived urban communities, findings may not be directly generalisable to CHWW programmes operating in different geographic, demographic or healthcare system contexts.

## Introduction

### Background and rationale

 Preventive healthcare is widely recognised as the foundation of an effective and equitable health system, enabling early detection, risk reduction and proactive management of disease to improve population health and reduce inequalities.^1–5^ Despite the universal coverage of the National Health Service (NHS) in England, the uptake of preventive services remains persistently unequal, with notably lower participation among groups living in deprived areas, ethnic minority communities and those with limited health literacy or unstable social circumstances.^6–8^ These inequalities undermine the sustainability of the NHS by contributing to higher rates of avoidable illness and hospitalisation, increasing the pressure on urgent and emergency services and driving declining vaccination coverage and delayed cancer detections.^7 9–15^

A range of interventions, such as national awareness campaigns and digital reminders, have been developed to improve preventive uptake.^16 17^ However, these traditional interventions have largely focused on changing individual behaviour or increasing awareness, often neglecting the structural, social and relational factors that shape a household’s ability to engage with preventive care.^16 17^ Evidence suggests that improving preventive health engagement requires moving beyond generic, short-term campaigns toward sustained, personalised and locally embedded community-based approaches.^18 19^

Community health workers (CHWs)—lay health workers recruited from local communities to deliver basic health promotion and navigation support—are widely regarded as a cost-effective complement to clinical professionals in overcoming these structural barriers.^19–25^ One of the most influential examples is Brazil’s Community Health Worker programme, established as part of the Family Health Strategy (FHS).^26 27^ By integrating trained, salaried CHWs into local primary care teams to conduct routine, proactive household visits, the FHS has achieved substantial population-level improvements, including increased vaccination and cancer screening uptake and significant reductions in infant and cardiovascular mortality.^27–33^

Drawing on the principles of the Brazilian FHS, Westminster City Council piloted a community health and well-being worker (CHWW) role in 2021. Grounded in universal coverage, comprehensive household support, hyperlocal engagement and system integration, CHWWs were assigned to defined household lists within a socioeconomically deprived area of central London to conduct regular, proactive monthly visits. An early observational evaluation of this pilot reported encouraging improvements in the uptake of immunisations, cancer screening and NHS Health Checks among visited households.^34^

Despite this global evidence and the promising early findings from the Westminster pilot, England has not adopted a scaled national CHW workforce. Empirically, there remains a critical evidence gap in establishing high-level, causal evidence on the effectiveness of CHWW interventions in the UK. Existing evidence within the UK context is currently limited to small-scale, non-randomised evaluations.^34^ Given growing policy interest and the ongoing expansion of the CHWW model into other UK regions, it is essential to generate robust empirical evidence to inform future implementation, scalability and integration within the NHS.

Building on the findings of the initial pilot, and spearheaded by the National Association of Primary Care (NAPC), the CHWW intervention has scaled into over 20 locations in England and has informed the NHS 10-year plan as a key workforce solution to deliver left-shift, integrated neighbourhood care.^35 36^ In Westminster, the pilot expanded across the most deprived areas of North and South Westminster, with a further 19 CHWW roles employed in January 2023 that were allocated at random by the lead primary care network (PCN) provider, Healthcare Central London (HCL) and integrated into routine primary care and community services. This paper describes the protocol for a pragmatic cluster randomised controlled trial, using routinely collected health data, to determine whether embedding CHWWs within routine care improves preventive healthcare uptake in deprived urban communities in England.

The overarching aim of this study is to evaluate the effectiveness of a scaled, proactive community-based intervention, compared with usual care, on improving the uptake of preventive care services among households in deprived areas of Westminster, London. Given the wide range of advice and support that CHWWs provide at the household level, we use a previously proposed composite measure that captures the uptake of all preventive healthcare services at the household level between intervention and control clusters, as measured by the Composite Referral Completion Indicator (CRCI).^37^ The secondary objective is to assess the effect of the intervention on patterns of healthcare use at the household level, specifically focusing on all-cause general practice (GP) attendances, accident and emergency (A&E) attendances, unplanned emergency hospital admissions and 30-day emergency hospital readmissions. These outcomes will be used to assess the potential downstream benefits of improved preventive care uptake, as well as unintended consequences such as increased pressure on GP or emergency services.

We hypothesise that households in intervention clusters will demonstrate a higher rate of preventive healthcare uptake, as measured by the CRCI, compared with control households.

### Methods and analysis

This protocol (V.2, June 2026) has been prepared in accordance with the Standard Protocol Items: Recommendations for Interventional Trials (SPIRIT) reporting guidelines. A completed SPIRIT checklist is provided in the online supplemental material. The planned reporting of the trial results will follow the Consolidated Standards of Reporting Trial (CONSORT).^38^

#### Study design and setting

This is a pragmatic, parallel-group, superiority cluster-randomised controlled trial with blinded outcome assessment. The trial evaluates the real-world impact of embedding CHWWs within primary care and community systems. The study is conducted in the London Borough of Westminster, specifically targeting geographically defined ‘village’ clusters located within the most socioeconomically deprived 20% of lower super output areas (LSOAs) nationally.

#### Eligibility criteria

Eligibility is defined at both the cluster and household levels.

#### Cluster/site level

Eligible clusters are geographically defined areas referred to as ‘villages’ within the CHWW programme—the pre-established operational units of intervention delivery—each comprising approximately 120 households located within the targeted LSOAs and covered by participating PCNs. Villages represent the natural and necessary unit of clustering for this trial. Each CHWW is assigned to a single village and operates within it universally, proactively engaging all households.

#### Household/individual level

All individuals residing within eligible village households who are registered with a GP that is part of the HCL PCN, contributing to the routine healthcare dataset are included, regardless of age, health status or healthcare engagement. Households lacking valid address identifiers for data linkage are excluded.

#### Intervention: community health and well-being worker model

The intervention consists of a comprehensive, proactive, community-based service, designed to address health and social needs across the life course. The CHWW model is embedded within routine primary care and community services and is delivered at the household level.

#### Intervention providers

CHWWs are lay health workers locally recruited by commissioning organisations who have completed standard competency training prior to deployment. The role is intentionally broad and holistic, encompassing health, well-being and wider social determinants of health.

#### Intervention arm (usual care+CHWW model)

CHWWs provide proactive (ie, no referral needed, door-knocking approach), comprehensive household outreach primarily through face-to-face visits, aiming for monthly contact. Core activities include promoting the uptake of preventive care (immunisations, cancer screenings, NHS Health Checks), addressing social determinants of health and navigating referrals to clinical and community services. The intensity and duration of contact are tailored to household needs.

### Key features of delivery include

- Frequency: CHWWs aim to visit, or attempt to visit, each household in their assigned village at least once per month, with more frequent contact where needs are identified.
- Approach: All households in intervention villages are approached, irrespective of demographic characteristics, clinical history or prior service use.
- Mode of delivery: In-person visits are supported by a digitally enabled application, which provides reminders and interactive educational materials.
- Continuity: Regular contact enables follow-up of identified concerns, monitoring of progress and timely response to new issues.

Full intervention description in accordance with the Template for Intervention Description and Replication (TIDieR) checklist is provided in online supplemental material 2.

### Training, materials and standardisation

All CHWWs complete a standard training programme specified by the commissioning organisations prior to deployment. CHWWs are recruited based on their motivation for the role, residency in or close to the local neighbourhood, emotional intelligence and commitment to helping others. No prior professional qualification is required beyond secondary school education. Following recruitment, CHWWs complete 3–4 weeks of training covering online resources, just listening, data management, confidentiality, lone working, safeguarding and common health conditions. On-the-job support is provided through weekly clinical supervision sessions with a lead GP, discussion of complex cases and identification of learning opportunities. Reflexive practice is supported through case write-ups and vignettes reviewed with line managers. Note: a CHWW level 3 certificate and apprenticeship has since become available through the Royal Society of Public Health to support scaling of the role nationally, but this was not a requirement during the intervention period of this study.

CHWWs operate under standard operational guidance and service protocols developed by the provider organisations. These materials support routine service delivery and staff training. They are not trial-specific and are available on request from the NAPC.

#### Comparator arm (usual care)

Households in control clusters receive standard preventive healthcare and community services (eg, routine national invitations and opportunistic delivery of screening and immunisations).

The CHWW intervention is delivered in addition to usual care, and no concomitant care is prohibited in either arm.

Given the pragmatic design, there are no trial-mandated criteria for discontinuing or enforcing the intervention. Adherence and intervention delivery will be assessed descriptively using routine operational data (the Octolink application) that CHWWs use to monitor their productivity.

### Outcomes and data source

All outcomes are derived from the Whole Systems Integrated Care (WSIC) database, which integrates electronic health records across Northwest London. The WSIC platform comprises linked, pseudonymised health service data from Northwest London.^39^ Data are accessible via a remote server within a secure Trusted Research Environment hosted by Imperial College Health Partners UK. Access is granted solely through an approved application process and is subject to information governance approvals; all analyses are conducted within this environment, and no identifiable data are extracted. Record linkage across data sources is performed by the WSIC team.

### Primary outcome

The CRCI is the primary outcome developed specifically for this intervention in 2015.^37^ However, it was adapted to a rate outcome to accommodate the eligibility nature of its components. In this study, it is defined as the household-level rate of completed, eligible preventive healthcare services per person-year. It encompasses age-specific and sex-specific immunisations, NHS Health Checks and bowel, breast and cervical cancer screenings. The primary outcome CRCI was selected to capture overall uptake of preventive healthcare services across multiple domains. A composite outcome was considered appropriate because the CHWW model is designed as a holistic intervention that addresses multiple preventive care needs simultaneously rather than targeting a single condition or service. The CRCI also reflects the real-world and comprehensive nature of the intervention. The components of the CRCI are summarised in table 1.

**Table 1:** Preventive services included in the composite primary outcome CRCI

Preventive service	NHS eligibility criteria*	Definition of completion
Adult immunisations		
Influenza	Adults aged ≥65 years	Receipt of influenza vaccination during an eligible vaccination season.
COVID-19 booster	Adults aged ≥75 years	Receipt of a COVID-19 booster vaccination during an eligible vaccination season.
Pneumococcal	Adults aged ≥65 years	Receipt of pneumococcal vaccination within study period.
RSV	Adults aged ≥75 years	Receipt of RSV vaccination within study period.
Shingles	Adults aged 70–79 years	Receipt of shingles vaccination within study period.
Childhood immunisations		
Influenza vaccination	Children aged 2–15 years	Receipt of influenza vaccination during an eligible vaccination season.
6-in-1	First dose: Children aged 8 weeks	Receipt of the scheduled vaccine dose within study period.
	Second dose: Children aged 12 weeks	
	Third dose: Children aged 16 weeks	
MMR	First dose: Children aged 12 months.	Receipt of the scheduled vaccine dose within study period.
	Second dose: Children aged 18 months or 3 years and 4 months.	
Rotavirus	First dose: Children aged 8 weeks Second dose: Children aged 12 weeks	Receipt of the scheduled vaccine dose within study period.
Meningitis B	First dose: Children aged 8 weeks	Receipt of the scheduled vaccine dose within study period.
	Second dose: Children aged 12 weeks Third dose: Children aged 12 months to 2 years	
4-in-1 pre-school booster	Children aged 3 years and 4 months to 5 years	Receipt of the scheduled vaccine dose within study period.
Td/IPV adolescent booster (3-in-1)	Children aged 13 or 14	Receipt of the scheduled vaccine dose within study period.
MenACWY	Children aged 13 or 14	Receipt of the scheduled vaccine dose within study period.
HPV	Children aged 12–13 years	Receipt of the scheduled vaccine dose within study period.
Cancer screening		
Cervical cancer screening	Women 25–64 years, every 5 years	Receipt of service within study period.
Breast cancer screening	Women 50–71, every 3 years	Receipt of service within study period.
Bowel cancer screening	Men and women 50–74 years, every 2 years	Receipt of service within study period.
Preventive health assessments		
NHS Health Check	Men and women 40–74 years, every 5 years	Receipt of service within study period.

* Detailed operational definitions, including age calculations, eligibility windows, screening due dates, exclusion criteria and coding algorithms, will be specified in the Statistical Analysis Plan.

CRCI, Composite Referral Completion Indicator; HPV, Human Papilloma Virus; IPV, Inactivated Polio vaccine; MenACWY, Meningitis ACWY; MMR, Measles/Mumps/Rubella; NHS, National Health Service; RSV, Respiratory Syncytial Virus; Td, Tetanus and Diphtheria.

### Eligibility for preventive services

Eligibility for preventive services will be assessed dynamically throughout the study. Participants may become eligible for one or more preventive services at any point between 1 January 2024 and 31 December 2025 according to NHS eligibility criteria. For each preventive service, participants will enter the risk set on the date they first become eligible. No new eligibility episodes will be accrued after 31 December 2025.

Once eligible, participants will remain under follow-up until completion of the preventive service, death, or administrative censoring on 30 June 2026, whichever occurs first. Preventive service uptake occurring between 1 January 2026 and 30 June 2026 will therefore contribute to the outcome if eligibility was established on or before 31 December 2025. The conceptual framework for eligibility accrual and follow-up is presented in table 2.

**Table 2:** Conceptual framework for eligibility accrual and follow-up for the CRCI

Components	Definition
Intervention period	January 2024 to December 2025. Period during which CHWWs deliver the intervention.
Eligibility accrual period	January 2024 to December 2025. Participants may become eligible for one or more preventive services according to NHS criteria during this period. No new eligibility episodes are accrued after 31 December 2025.
Eligible opportunity	A preventive service for which a participant meets NHS eligibility criteria and has not yet completed the service.
Entry into risk set	Date on which a participant first becomes eligible for a preventive service.
Outcome	Completion (uptake) of the recommended preventive service.
Follow-up period	From entry into the risk set until service completion, death or administrative censoring, whichever occurs first.
Administrative censoring date	30 June 2026.
Seasonal services	Influenza vaccination may contribute more than one eligible opportunity because eligibility is renewed for each vaccination season.
Repeat eligibility after uptake	Following completion of a preventive service, participants exit the risk set for that service. Re-entry is not expected for screening services and NHS Health Checks during the study period.

CHWW, community health and well-being worker; CRCI, Composite Referral Completion Indicator; NHS, National Health Service.

### Secondary outcomes

1. Uptake rates (events per person-year) for each individual preventive service component (immunisations, NHS Health Checks and bowel, breast and cervical cancer screenings).
2. Household-level healthcare utilisation rates or counts, specifically all-cause GP attendances, A&E attendances, unplanned hospital admissions and 30-day hospital readmissions.

### Participant timeline

Villages were randomised prior to CHWW deployment, with both occurring before 1 January 2024. The household intervention commenced on 1 January 2024, with CHWW training continuing on an ongoing basis throughout the first 6 months of 2024. The eligibility window for preventive services ran until 31 December 2025, with final follow-up and outcome assessment concluding on 30 June 2026 (see table 3).

**Table 3:** Schedule of enrolment, intervention and assessments

	Trial period					
	Enrolment		Post-randomisation			Follow-up
Time point^B^	−t_i_	0	Month 1	Month 6	Month 24	Month 30
Enrolment:						
Eligibility screen	X					
Randomisation	X					
CHWW training		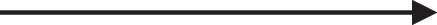				
Intervention/CHWW:			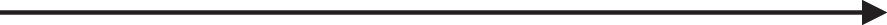			
Assessments:						
Demographics					X	X
Preventive services uptake					X	X
Healthcare use					X	X

CHWW, community health and well-being worker.

### Sample size and power calculation

The sample size for this trial is determined primarily by operational and service delivery capacity, with approximately 60 village clusters mapped within the eligible deprived areas. These 60 clusters (comprising roughly 7200 households) were randomised in a 1:2 ratio (2280 intervention; 4920 control).

### Initial calculation and methodological adjustments

The initial sample size estimate registered on ClinicalTrials.gov treated the primary outcome as a simple proportion. The trial was designed to provide >90% power (2α=0.05) to detect an absolute improvement of 10% in the uptake of preventive services assuming a within-period intraclass correlation coefficient (ICC) of 0.15 and baseline uptake of 15% under usual care conditions (demonstrated from pilot evaluation). This calculation assumed a ‘two-period decay’ thus allowing for the within-cluster correlation within the same measurement period to differ to that from a different measurement period. The within-cluster correlation from different periods was assumed to be 80% of the correlation from the same period. For repeated within-household measurements, it was assumed to be a correlation of 0.5. Finally, this calculation assumed that at least 120 households will be included in each village, thus resulting in a total of at least 7200 households (2280 randomised to the intervention and 4920 randomised to usual care). Under these assumptions, the 60 available clusters will provide at least 90% power to evaluate the intervention’s effect on the CRCI, using a two-sided significance level of 0.05. Power calculation was conducted using an online cluster randomised controlled trial calculator (https://clusterrcts.shinyapps.io/rshinyapp/). However, the primary outcome (CRCI) is more accurately evaluated as a rate-based outcome measuring the uptake of preventive services over varying lengths of follow-up (person-years). Because standard sample size calculators do not natively handle varying person-years, the power calculation will be updated to use a more precise methodology.

### Updated power calculation

The achieved statistical was re-estimated using the closed-form formula derived by Li *et al* based on generalised estimating equations (GEE).^40^ This formula directly accommodates the trial’s pragmatic features: count/rate outcome, unequal cluster sizes, overdispersion, varying person-years and an unequal 1:2 randomisation ratio. Since the total number of clusters (N=60) is fixed by deployment, this re-estimation solves for achieved power at the observed sample size rather than calculating a required sample size.

The nuisance parameters (ICC, overdispersion, cluster-size moments and follow-up moments) were re-estimated from January 2024 to December 2025 interim trial data rather than from the pre-trial historical WSIC data originally planned. This update represents the actual enrolled households in the 60 trial clusters, making it the most accurate reflection of the study population. The treatment effect parameter (β_20_=log(0.25/0.15) ≈ 0.511) was kept fixed at the protocol-specified value throughout and was never re-estimated from interim data. This is a critical safeguard against type I error inflation, which is consistent with the nuisance parameter only recalculation framework described by Friede and Schmidli, Harden and Friede.^41 42^

Under these parameters, the trial achieves power >90% for N=60 clusters to detect an absolute increase of 0.10 CRCI events per person-year (from 0.15 to 0.25), at a two-sided significance level of 0.05. Full details of the parameter’s estimation, sensitivity analyses and methodological justification are provided in the Statistical Analysis Plan (SAP) as online supplemental file 3.

### Assignment of interventions

The 60 villages will be randomised in a 1:2 ratio (intervention: control). An independent statistician will generate the allocation sequence using a computer-generated algorithm, stratified by geography (North vs South Westminster) and regional CHWW allocation. Due to the visible nature of the intervention, residents and CHWWs cannot be blinded; however, the analysts assessing the routine WSIC data will remain blinded to allocation until the SAP is finalised. No individual-level recruitment or consent is undertaken.

### Data management

Pseudonymised outcome data will be extracted from the WSIC database at predefined time points. Specifically, a fixed point-in-time cohort will be defined at the trial start (1 January 2024) to establish baseline eligibility and minimise survivorship bias. Participant follow-up ends at the earliest occurrence of service completion, death or the end of the study period (30 June 2026). Missing outcome data (eg, no record of a completed screening) will be treated as service non-completion, reflecting standard administrative data practices.

### Statistical methods

All analyses will follow a prespecified SAP. The primary analysis will be conducted according to the intention-to-treat principle. All eligible households and individuals will be analysed according to the original randomised allocation of their village, irrespective of engagement with the CHWW intervention.

Baseline characteristics will be summarised descriptively at village, household and individual levels. Further details are provided in SAP as online supplemental file 3.

### Primary outcome analysis

The CRCI treatment effect will be estimated at the household level using a generalised linear mixed model (GLMM) with a Poisson family distribution, log link and an offset for log(person-years). The model includes a random intercept for village (cluster) and fixed effects for treatment arm and randomisation stratum (North vs South). The model will adjust for prespecified baseline covariates: household size. The effect will be expressed as an adjusted incidence rate ratio with a 95% CI.

If overdispersion is detected (variance substantially exceeding the mean, assessed via Pearson residual deviance and likelihood ratio tests), the analysis will use a mixed-effects negative binomial regression as an alternative. If the mixed-effects model fails to converge, a GEE with exchangeable correlation structure and robust sandwich SEs will be fitted.

#### Household composition

Household membership will be defined at and fixed to 1 January 2024. Preventive service eligibility and uptake will be based on household members present at that date. Individuals contribute person-time and outcomes only while registered and under observation (until death or study end). Household denominators will therefore reflect the aggregated observation time of study cohort, consistent with at-risk approach planned for this trial.

### Secondary outcome analysis

Secondary outcomes will be analysed using a similar GLMM framework. However, the results of secondary outcomes will be presented as effect estimates with 95% CIs. These analyses are exploratory and hypothesis-generating, results will be interpreted with caution and without formal multiplicity correction.

Absolute effects: To inform public health policy, the absolute rate difference will be estimated as a prespecified exploratory analysis using cluster-level hierarchical targeted maximum likelihood estimation, which robustly handles varying person-time and missing-at-random right-censoring in cluster trials.^43^

Sensitivity analyses: Prespecified sensitivity analyses include a per-protocol analysis defined as only including participants who actively engaged with a CHWW and were coded as such in the clinical records, and an evaluation of a 12-month (rather than 24-month) pre-trial baseline covariate adjustment to account for potential post-COVID-19 pandemic distortions in service uptake.

Analyses will use two-sided tests with a significance level of 0.05. Analyses will be conducted using the latest R software.

### Trial monitoring and harms

Given the pragmatic, low-risk nature of the intervention and the use of retrospective, anonymised data, a formal Data Safety Monitoring Committee is not required. No clinical adverse events are anticipated; unintended harms (eg, safeguarding concerns or issues) fall outside the study team’s remit, however, CHWW programme managers will notify the study team of any relevant adverse events. One descriptive interim analysis will be conducted in April 2026 for oversight purposes, with no formal hypothesis testing or stopping guidelines applied.

### Patient and public involvement

Patients and members of the public were not formally involved in the methodological design of the randomised evaluation or the selection of statistical outcomes. However, the CHWW intervention itself was designed and scaled in direct response to locally identified needs and ongoing community feedback from the earlier Westminster pilot. This highlights the vital importance of public partner involvement—championed throughout the National Institute for Health and Care Research Northwest London Applied Research Collaboration—ensuring the model remains firmly grounded in residents’ lived experiences. Furthermore, because CHWWs are recruited directly from their own communities, they provide continuous public engagement during routine service delivery. Study findings will be disseminated to local stakeholders and participating communities through plain-language summaries.

## Ethics and dissemination

### Research ethic approval and consent

Formal individual participant consent is not required for this study. Because the CHWW intervention is a new routine service being offered by the PCN, engagement with the service implies consent for the care provision itself. Following advice from the Imperial College London Research Ethics Committee (REC), Health Research Authority (HRA) approval was sought in lieu of a separate REC ethical review. Furthermore, formal research consent to access and analyse the data is not needed because it falls under the existing global ethics approval for research use of WSIC dataset in Northwest London (REC reference: REC18/WM/0323/IRAS252449). The study will be conducted in accordance with the Declaration of Helsinki.

### Confidentiality and data management

All pseudonymised data will remain within the secure WSIC environment, and no identifiable information will be extracted. Only aggregate results (eg, tables, statistical models and figures) will be exported for publication. Access to the dataset is strictly restricted to approved members of the research team under WSIC information governance arrangements. The chief investigator is registered under the Data Protection Act to ensure confidentiality is preserved. A formal Data Protection Impact Assessment was not required as the planned analyses do not meet the criteria for high-risk processing of personal data.

### Ancillary care and safety

Given the pragmatic, low-risk and non-pharmacological nature of the CHWW intervention, no trial-specific ancillary or post-trial clinical care is planned. Participants will continue to access routine NHS and social care services throughout the trial. Any safeguarding concerns, issues or serious incidents identified during routine CHWW activities will be managed strictly through the existing organisational governance and safeguarding procedures of the local commissioning providers (Healthcare Central London, Westminster City Council and One Westminster). No financial compensation is provided for trial participation.

### Protocol amendments

Any important modifications to the study protocol (eg, changes to eligibility criteria, outcomes or statistical analyses) will be documented and communicated to the research team, HRA/WSIC governance, and the trial registry.

### Dissemination policy

The findings of this evaluation will be disseminated through peer-reviewed academic publications, conference presentations and policy documents. In addition, plain-language reports will be shared with local stakeholders and participating communities. A formal publication protocol will guide authorship eligibility and the dissemination process.

## Supplementary material

10.1136/bmjopen-2026-124582 online supplemental file 1

10.1136/bmjopen-2026-124582 online supplemental file 2

10.1136/bmjopen-2026-124582 online supplemental file 3
