# Supplementary material for: Community health and well-being workers (CHWWs) for preventive healthcare services uptake in the UK: a cluster-randomised controlled trial protocol
Source: BMJ Open. 2026 Oct 23;16(10):e124582. doi: 10.1136/bmjopen-2026-124582 (PMC13711556; doi:10.1136/bmjopen-2026-124582)
Supplement: Supplementary file 1 [file bmjopen-16-10-s002.pdf]

TIDieR

Community Health and Wellbeing Worker (CHWW)

**Why:**	To address health and social needs across the life course by improving uptake of preventive care and targeting wider social determinants of health, embedded within routine primary care.
**What (material):**	CHWWs use the Octolink digital application during household visits, which provides visit scheduling reminders and records the activity of CHWWs. Octolink access is for registered users only, the link to platform can be found here: https://octoapp.co.uk/. CHWWs also operate according to standard operational guidance and service protocols developed by the commissioning organisations; these materials are not trial-specific and are available upon request from the National Association of Primary Care (NAPC).
**What (procedures):**	Proactive, unsolicited door-knocking outreach to all households in intervention villages; promoting uptake of preventive services (immunisations, cancer screenings, NHS Health Checks); addressing social determinants of health; navigating and facilitating referrals to clinical and community services; follow-up of identified needs.
**Who provided:**	CHWWs, lay health workers locally recruited by commissioning organisations and trained in core competencies. CHWWs are recruited based on their motivation for the role, residency in or close to the local neighborhood, emotional intelligence, and commitment to helping others. No prior professional qualification is required beyond secondary school education. Following recruitment, CHWWs complete three to four weeks of training covering online resources, Just Listening, data management, confidentiality, lone working, safeguarding, and common health conditions. On-the-job support is provided through weekly clinical supervision sessions with a lead GP, discussion of complex cases, and identification of learning opportunities. Reflexive practice is supported through case write-ups and vignettes reviewed with line managers.
**How (mode of delivery; individual or group):**	Primarily face-to-face in-person household visits, supported by the Octolink digital application.
**Where:**	Participants' homes within intervention villages (located within the most socioeconomically deprived 20% of Lower Super Output Areas (LSOAs) nationally), London Borough of Westminster.
**When and how much:**	At least monthly contact per household; more frequent contact where greater needs are identified; intensity and duration of each visit tailored to household needs; delivered throughout the intervention period (1 January 2024 - 31 December 2025)
**Tailoring:**	Frequency and duration of contact adapted to individual household needs; all households approached regardless of demographic characteristics, clinical history or prior service

	use. Community Health and Wellbeing Worker (CHWW)
**How well (planned):**	Fidelity will be assessed by examining WSIC and Octolink data to ascertain the proportion of households allocated to the CHWW intervention that received at least one CHWW visit during the intervention period.
