# Supplementary material for: Community health and well-being workers (CHWWs) for preventive healthcare services uptake in the UK: a cluster-randomised controlled trial protocol
Source: BMJ Open. 2026 Oct 23;16(10):e124582. doi: 10.1136/bmjopen-2026-124582 (PMC13711556; doi:10.1136/bmjopen-2026-124582)
Supplement: Supplementary file 2 [file bmjopen-16-10-s003.pdf]

Statistical analysis plan for the Community Health and Wellbeing Worker (CHWW) trial on the uptake of preventive healthcare services

Authors:
Authors:
Rawan Gadi[1,2], Connie Junghans[1,3], Ahmad Alkhatib[1], Laurent Billot[4], Thomas Woodcock[1], David Peiris[4], Otavio Berwanger[4], Matthew Harris[1]

Affiliations:

1. Department of Primary Crae and Public Health, School of Public Health, Imperial College London, London, UK.
2. Dental Public Health Department, Faculty of Dentistry, King Abdulaziz University, Jeddah, Saudi Arabia.
3. Geller Institute for Aging and Memory, University of West London, London, UK.
4. The George Institute for Global Health, Faculty of Medicine and Health, UNSW Sydney, Sydney, Australia

Abstract:
The Community Health and Wellbeing Worker (CHWW) trial aims to determine whether proactive, community-based outreach delivered by trained community health and wellbeing workers increase the uptake of preventive healthcare services compared with usual care in deprived urban areas of Northwest London. It is designed as a pragmatic, two-arm, parallel, cluster-randomised controlled trial with 60 villages randomised in a 1:2 ratio, using the household as the primary unit of analysis. This statistical analysis plan outlines the predefined analytical methods for all trial outcomes and key variables to ensure transparency and reproducibility.

The primary outcome is the Composite Referral Completion Indicator (CRCI), defined as the rate of completed preventive services per person-year of time at risk, aggregated across immunisation, cancer screening, and NHS Health Check services over the combined 2024-2025 period.

The primary analysis will use a mixed-effects Poisson regression adjusted for clustering by village using random intercepts, and for region and trial arm as fixed effects. A log(person-time) offset will be included to account for differences in observation time. The effect of CHWW intervention will be estimated as rate ratio with its corresponding 95% confidence interval,

Sensitivity analyses will include covariate adjustments and checks of model assumptions. Additional analyses will test the robustness of results to different follow-up definitions to assess the consistency and timing of intervention effects.

Keywords: community health worker, clinical trial, cluster randomised trial, primary care, preventive services uptake, statistical analysis plan.

Table of Contents

1 Administrative information ... *4*

2 Introduction ... *5*

2.1 Study synopsis ... 5

2.2 Study population ... 5

2.3 Study interventions ... 6

2.4 Randomisation and blinding ... 7

2.5 Outcomes ... 7

2.6 Statistical hypotheses ... 13

2.7 Sample size ... 13

3 Statistical analysis ... *14*

3.1 Statistical principles ... 14

3.2 Datasets analysed ... 15

3.3 Subject disposition ... 15

3.4 Handling of protocol deviations ... 15

4 Planned analyses ... *15*

4.1 Baseline characteristics and comparisons ... 15

4.2 Analysis of the primary outcome ... 16

4.3 Analysis of secondary outcomes ... 18

5 References ... *19*

6 Appendix ... *20*

1 Administrative information

1.1 study identifiers: (Please can you fill in the required here)

- Protocol number/version: 0.3 Date: 9 june 2026
- ClinicalTrial.gov register Identifier/ UK trial registry: NCT06144580

1.2 Revision history

Version	Date	Details
0.1 (draft)	24 September 2025	First draft by RG, sent to MH,LB,TW,AA for review.
0.2	29 September 2025	Revised by MH,LB,AA,TW , following comments from LB, and TW. Confirmed change to rate ratio for primary analysis.
0.3	6 October 2025	Reviewed by MH and LB. following comments from MH and LB. Consolidated by LB.
0.2	20 May 2026	Including final table shells, adding references. Version ready for review for final version
0.3	June 2026	Formatted for submission

1.3 Contributors to the statistical analysis plan

1.3.1 Roles and responsibilities

Name	Affiliation	Role on study	SAP contribution
**Rawan Gadi**	Imperial College London, School of Public Health, London, UK. King Abdulaziz University, Faculty of Dentistry, Jeddah KSA.	SAP primary author	Developed and wrote the statistical analyses plan.
**Laurent Billot**	The George Institute for Global Health, Faculty of Medicine and Health, UNSW Sydney	Study statistician	Reviewed every version.
**Ahmad Alkhatib**	Imperial College London, School of Public Health, London, UK.	SAP contributor	Reviewed every version.
**Thomas Woodcock**	Imperial College London, School of Public Health, London, UK.	SAP contributor	Reviewed every version.
**Cornelia Junghans Minton**	Imperial College London, School of Public Health, Geller Institute for Aging and Memory,	SAP contributor	Reviewed every version.

	University of West London		
**David Peiris**	The George Institute for Global Health, Faculty of Medicine and Health, UNSW Sydney, Australia	SAP contributor	Reviewed every version.
**Otavio Berwanger**	The George Institute for Global Health, Faculty of Medicine and Health, UNSW Sydney, Australia	SAP contributor	Reviewed every version.
**Matthew Harris**	Imperial College London, School of Public Health, London, UK.	SAP contributor	Reviewed every version.

1.3.2 Approvals

Name	Signature	Date
Rawan Gadi	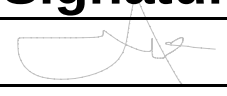	16 June 2026
Matthew Harris		16 June 2026
Laurent Billot		

2 Introduction

2.1 Study synopsis

The CHWW trial is a pragmatic, prospective, two-arm, parallel, cluster-randomised, blinded outcome-assessed study designed to evaluate whether the Community Health and Wellbeing Worker (CHWW) model increases the uptake of preventive healthcare services compared to usual care in deprived urban settings in Northwest London. In this trial, the unit of randomisation is the village (defined as a geographically bounded neighbourhood). A total of 60 villages were randomised in a 1:2 ratio, with 19 allocated to the CHWW intervention and 41to the control arm. The study commenced on 1 January 2024 and will run until 31 December 2025, with a 6-month follow-up period until 30 June 2026 to allow for the behavioural and system-level impacts of the intervention to be fully captured. Preventive service outcomes, including cancer screening, immunisations, and NHS Health Checks, will be assessed using the Composite Referral Completion Indicator (CRCI). Although the trial evaluation is restricted to the study period, the CHWW model has been explicitly cited in the NHS Long Term Plan as a potential solution for delivering integrated neighbourhood-level healthcare, highlighting its anticipated long-term role beyond the immediate study timeframe.

Villages allocated to the intervention arm are served by trained, paid CHWWs who each cover approximately 120 households in defined geographies or neighbourhoods. CHWWs conduct proactive monthly visits irrespective of demand, providing comprehensive support to households and facilitating uptake of preventive services. Villages in the control arm continue to receive usual care without CHWWs.

The trial is designed to evaluate the impact of the CHWW model on the, a household-level measure capturing uptake of recommended preventive services. In addition, service-specific outcomes and patterns of healthcare utilisation (GP attendances, emergency department visits, and hospital admissions) will be analysed to provide a fuller understanding of the intervention's effects. The full protocol is currently registered on clinicalTrials.gov (Identifier: NCT06144580). Patient follow-up will be completed by 30 June 2026, and the study closed by 30 September 2026. The main results will be published in December 2026.

2.2 Study population

The study population comprises households and individuals residing in 60 villages (clusters) in Northwest London. These villages represent defined geographical neighbourhoods and were selected in collaboration with local authorities and research team members. The trial uses households as the primary unit of analysis, with individual-level data used to define household eligibility for preventive care outcomes.

Eligibility for analysis of the primary outcome is defined at the household level, based on the presence of at least one individual eligible for preventive care services during the study period. On the other hand, secondary outcomes include all households in the trial as these outcomes do not depend on specific preventive care criteria.

2.2.1 Definition of the trial population:

The study population comprises households and individuals residing in 72 60 'villages' (clusters) in Northwest London. These villages represent defined geographical neighbourhoods and were selected in collaboration with local authorities and research team members. The trial uses households as the primary unit of analysis, with individual-level data used to define household eligibility for preventive care outcomes.

The trial population will be defined as all individuals who were resident and registered with a general practice (GP) on 1 January 2024, representing the start of the observation period. Households are defined as groups of individuals sharing the same residential address at this date.

New entrants after 1 Jan 2024 will not be included in the analytic cohort. Their presence will be reported descriptively but they will not contribute to the trial outcomes. Although the study area is characterised by high residential turnover, with individuals moving in and out frequently, for feasibility, household composition is defined at the start of the study and treated as fixed.

However, the earliest available extract from the Whole System Integrated Care (WSIC) for the trial is based on November 2024. This dataset will serve as the source dataset to identify the study participants. Specifically, the extract will be used to reconstruct the population registered at the start of 2024 by selecting individuals with active registration on 1 January 2024 and excluding those who had deregistered or died before that date. Some individuals registered on 1st January 2024 may have died or deregistered before November 2024 and will therefore be included in the study cohort but only contribute till the date of death or deregistration. This approach may slightly under-represent the true population at the start of 2024, but it provides the earliest complete dataset available and remains consistent with the operational rollout of the intervention as CHWWs were in training and preparatory phases until June 2024, with household visits commencing later in the year. The study cohort derived from the November extract, therefore, represents the earliest available comprehensive and reliable population snapshot from which to define the study population, while maintaining alignment with the true start of the trial period.

2.2.2 Follow-up and Censoring

Each individual will be observed from 1st January 2024 until the earliest of (i) death, (ii) service completion, or (iii) study end (30 June 2026).

At the household level, outcomes will be calculated by aggregating across members who remain under observation. A household will contribute outcomes as long as at least one baseline member is still under observation. The household follow-up will end only when all members have been censored or at study end, whichever comes first.

Outcomes will only be assessed for individuals who are actively registered in WSIC during their outcome assessment period. Those who deregister or die contribute outcomes to their censoring date.

2.2.3 Inclusion criteria for the study participants:

The target cohort was identified by Healthcare Central London (HCL), using participants' residential address, deprivation index, and registered GP practice.

Eligibility criteria were applied at the trial start date (1 January 2024). Specifically, residents were eligible if they:

- Were registered with a GP in Northwest London, and
- Lived in a postcode located within a Lower Super Output Area (LSOA) ranked in the most deprived 20% of the national deprivation index, and
- Were residents in any of the randomised clusters.

2.2.4 Exclusion criteria:

households or individuals were excluded if they:

- Were not residents in any of the randomised clusters and,
- Did not meet the GP registration and LSOA-based deprivation criteria.

2.3 Study interventions

2.3.1 Intervention arm

Villages allocated to the intervention arm are served by CHWWs. CHWWs are lay people, trained and paid, and fully integrated into the primary care team. Each CHWW covers approximately 120 households, providing proactive monthly visits to all households within their assigned village, regardless of expressed need or demand.

The intervention is characterised by four pillars:

1. **Integration with the health system:** CHWWs are embedded within general practices and local authorities, ensuring coordination with primary care and community services.
2. **Universal outreach:** CHWWs visit all households in their assigned area, not just those actively seeking care.
3. **Comprehensive support:** CHWWs provide support across health and social domains, including facilitating uptake of preventive services such as immunisations, cancer screenings, and NHS Health Checks.

4. **Hyperlocal delivery:** CHWWs are recruited from and embedded in the communities they serve, ensuring trust and local engagement.

The proactive approach of CHWWs involves both direct facilitation of preventive care uptake and broader support to address barriers to accessing healthcare services.

2.3.2 Control arm

Villages allocated to the control arm continue with usual care. Residents in these areas have access to the preventive health services through standard NHS pathways, but without the proactive household visits or support provided by CHWWs. No additional intervention is introduced in control villages during the study period.

2.3.3 Background standard of care

In both arms, participants remain eligible for all routine health and social services ordinarily available in their area. Clinical care and public health programmes are delivered according to NHS guidelines, independent of trial allocation. The only difference between the two arms is the presence or absence of the CHWW model.

2.4 Randomisation and blinding

Randomisation was conducted at the village (cluster) level using 1:2 allocation ratio (intervention: control). Allocation was stratified by geographic region (north and south) to ensure balance in the number of eligible villages and the distribution of CHWWs across regions. While this stratification improves geographic comparability, it does not formally control for potential contamination between intervention and control areas. CHWWs record interactions with individuals residing outside their assigned village, and these data may later be triangulated with the study dataset to assess the extent of cross-village interactions and potential contamination.

An independent statistician generated the randomisation sequence using a computer-based algorithm. The randomisation schedule was securely stored on the HCL server. Allocation was concealed until randomisation was complete, and only cluster representatives were informed of their assignment.

Given the nature of the intervention, blinding of participants and CHWWs was not possible, as the CHWW service became part of routine care in intervention villages. However, outcomes will be assessed using routinely collected data from the WSIC database, and the statistical analysis team will remain blinded to allocation until database lock, thereby reducing the potential for bias in outcome assessment.

For blinding, each cluster was assigned a numeric code that does not reveal its allocation. Only the WSIC team retains the allocation file linking codes to trial arms. The analysis team will therefore work with an anonymised dataset and will remain blinded to allocation throughout data processing and analysis, except for the interim analysis scheduled for mid 2026, where partial unblinding will be required to generate the necessary descriptive summary for primary and secondary outcomes.

This randomisation process provides fair and transparent method for distributing CHWWs during their expansion in Westminster, while also enabling a rigorous, large-scale clustered randomised trial capable of addressing selection and confounding bias.

2.5 Outcomes

2.5.1 Primary outcome

The primary outcome is the CRCI, defined as a rate per person-year at risk at the household level, aggregated across preventive service opportunities.

For household h over the trial period the CRCI is:

$$CRCI_h = \frac{\Sigma_{\mathrm{i}\in\mathrm{h}}\Sigma_{\mathrm{s}\in\mathrm{S}}\mathrm{Event}_{i,s}}{\Sigma_{\mathrm{i}\in\mathrm{h}}\Sigma_{\mathrm{s}\in\mathrm{S}}\mathrm{Time-at-risk}_{i,s}}$$

Where:

- i index household members.

- $s \in S$ is the set of preventive services (immunisations, cancer screening, NHS Health Check).
- $\text{Event}_{i,s}$ =1 if service s was completed by individual *i* within the follow-up window, and 0 otherwise.
- $\text{Time} - \text{at} - \text{risk}_{i,s}$ = the number of years for which individual *i* was eligible for service s.
- Eligibility begins at the index date when NHS criteria are met and ends when the service is complete or follow-up ends.
- If time-at-risk = 0 (i.e the individual was never eligible), the opportunity does not contribute to the denominator.
- Interpretation: CRCI expresses the rate of completed preventive services per person-year at risk. Because households may be eligible for multiple services in the same period, CRCI can exceed 1.
- Households with zero eligible person-time across the study period will be excluded from the primary analysis but described descriptively.

Example:
Table 1 provides a worked example of how the CRCI will be calculated for one household. The household consists of two members, each eligible for different preventive services. For each service, the time-at-risk reflects how long each member was eligible according to NHS criteria. Once the service is completed, eligibility for that specific service ends.
For service 1, only member A completed the service, resulting in one event over 1.3 years of total time-at-risk (rate 0.77 per person-year).
For service 2, both members completed the service (two events over 1.5 person-years, rate 1.33 per person-year). For service 3, only member B completed the service (one event over 1.7 person-years, rate 0.59 per person-year).
The overall household-level is calculated by summing all events (4 completions) and dividing by the total time-at-risk (4.5 person-years):

$$CRCI_h = \frac{4}{4.5} = \mathbf{0.89}$$

This value represents the average number of preventive services completed per person-year at risk within the household. Because households can be eligible for multiple services, CRCI values may exceed 1 when the number of completions is greater than the total person-years at risk.

Table 1:

**Household member**	**Service 1**		**Service 2**		**Service 3**		**Composite**		
	**Time-at-risk (years)**	**Event (Yes)**	**Time-at-risk (years)**	**Event (Yes)**	**Time-at-risk (years)**	**Event (Yes)**	**Time-at-risk (years)**	**Event (Yes)**	**Rate (PY)**
**Member A**	0.50	1	1	1	0.70	0	2.20	2	0.91
**Member B**	0.80	0	0.50	1	1	1	2.30	2	0.87
**Household total**	1.30	1	1.50	2	1.7	1	4.50	4	**0.89**

2.5.1.1 Operational eligibility definitions for the CRCI

Eligibility for each preventive service will be determined using operational algorithms derived from NHS programme guidance. Participants may become eligible at any point between 1 January 2024 and 31 December 2025. Eligibility will therefore be assessed dynamically throughout the study period rather than solely at baseline. Table 2 summarises the operational eligibility algorithms used to identify eligible opportunities for each preventive service included in the CRCI.

Table 2: Operational eligibility definitions for preventive services included in the CRCI.

Category	Preventive service	Operational eligibility definition
**Adult immunisations**	**Influenza**	Adults aged 65 years or older at the start of an eligible influenza vaccination season.
	**COVID-19 booster**	Adults aged 75 or older during an eligible vaccination season.
	**Pneumococcal (PPV)**	Adults aged 65 years or older and have not previously received PPV.
	**RSV**	Adults aged 75 years or older and have not previously received RSV.
	**Shingles**	Adults aged 70 – 79 years and have not previously received shingles.
**Childhood immunisations**	**Influenza**	Children aged 2–15 years during an eligible vaccination season
	**6-in-1 (1st dose)**<br>**6-in-1 (2nd dose)**<br>**6-in-1 (3rd dose)**	1st dose: Children aged 8 weeks<br>2nd dose: Children aged 12 weeks<br>3rd dose: Children aged 16 weeks
	**MMR (1st dose)**<br>**MMR (2nd dose)**	1st dose: Children aged 12 months<br>2nd dose: Children aged 18 months or 3 years and 4 months.
	**Rotavirus (1st dose)**<br>**Rotavirus (2nd dose)**	1st dose: Children aged 8 weeks<br>2nd dose: Children aged 12 weeks
	**Meningitis B (1st dose)**<br>**Meningitis B (2nd dose)**<br>**Meningitis B (Booster)**	1st dose: Children aged 8 weeks<br>2nd dose: Children aged 12 weeks<br>Booster dose: Children aged 12 months to 2 years
	**4-in-1 pre-school booster**	Children aged 3 years and 4 months to 5 years during the eligibility accrued period.
	**Td/IPV adolescent booster (3-in-1)**	Children aged 13 or 14 during the eligibility accrued period.
	**MenACWY**	Children aged 13 or 14 during the eligibility accrued period.
	**HPV**	Children aged 12 to 13 years during the eligibility accrued period.
**Cancer screening**	**Cervical Cancer Screening**	Women aged 25–64 years who become due or overdue for screening according to NHS screening intervals (every 5 years) and have no prior cervical cancer diagnosis before risk-set entry.
	**Breast Cancer Screening**	Women aged 50–71 years who become due or overdue for screening according to NHS screening intervals (every 3 years) and have no prior breast cancer diagnosis before risk-set entry.
	**Bowel Cancer Screening**	Adults aged 50–74 years who become due or overdue for screening according to NHS screening intervals (every 2 years) and have no prior bowel cancer diagnosis before risk-set entry.
**Preventive health assessment**	**NHS Health Check**	Adults aged 40–74 years who become due or overdue for an NHS Health Check (every 5 years) and do not meet NHS Health Check exclusion criteria before risk-set entry.

2.5.1.2 Time at risk and outcome events for the CRCI

Eligibility alone does not contribute to the primary outcome. Once a participant becomes eligible for a preventive service, they enter a service-specific risk set and contribute time at risk until service completion, death, or administrative censoring. Because participants may become eligible at different points during the study period, risk-set entry is defined dynamically. Table 3 summarises the rules used to construct time at risk and outcome events for each category of preventive service.

Table 3: Time at risk and outcome events for the CRCI

Eligibility category	Included services	Risk-set entry date	Event definition	Censoring	Re-entry permitted
**Seasonal immunisations**	Influenza vaccination	Start of the relevant influenza season for individuals already eligible at season start, or the date eligibility criteria are first met during the season	Receipt of influenza vaccination during the relevant season	End of the influenza season or death, whichever occurs first	Yes. Each influenza season constitutes a separate eligible opportunity. Participants who remain eligible may re-enter the risk set in a subsequent influenza season.
**Programme-based adult immunisations**	COVID-19 booster, PPV, RSV vaccination, shingles vaccination	Later of: date programme eligibility criteria are met or 1 January 2024	Receipt of the relevant vaccination	Death or administrative censoring on 30 June 2026	No
**Age-triggered childhood immunisations**	6-in-1, MMR, Rotavirus, Meningitis B, 4-in-1 booster, Td/IPV booster, MenACWY, HPV	Later of date the child reaches the eligible age or 1 January 2024	Receipt of the corresponding vaccine dose	Death or administrative censoring on 30 June 2026	No
**Due-date-triggered screening programmes**	Cervical, breast, and bowel cancer screening	If already due or overdue on 1 January 2024: 1 January 2024. Otherwise: date screening becomes due	Completion of the corresponding screening programme	Death or administrative censoring on 30 June 2026	No
**Due-date-triggered preventive**	NHS Health Check	If already due or overdue on		Death or administrative	No

**health assessment**		1 January 2024: 1 January 2024. Otherwise: date NHS Health Check becomes due	Completion of an NHS Health Check	censoring on 30 June 2026	

Influenza vaccination was treated differently from other preventive services because eligibility recurs across seasonal vaccination campaigns. Each influenza season (1 September–31 March) was considered a distinct eligible opportunity. Participants who remained eligible could therefore contribute up to two influenza vaccination opportunities during the study period: one during the 2024/25 season and one during the 2025/26 season.

2.5.1.3 Service specific exclusion criteria

Service-specific exclusion criteria will be assessed before risk-set entry. Individuals meeting an exclusion criterion before the risk-set entry date will not contribute an eligible opportunity for the corresponding preventive service. For participants who are already due or overdue on 1 January 2024, exclusions will be assessed before this date. For participants who become eligible during the study period, exclusions will be assessed before the service-specific risk-set entry date (Table 4).

Table 4: Service specific exclusion criteria applied before risk-set entry

Preventive service assessment date	Exclusion criterion	Exclusion assessment date
**Cervical cancer screening**	Diagnosis of cervical cancer before risk-set entry	Before 1 January 2024 if already due/overdue; otherwise before the date screening becomes due
**Breast cancer screening**	Diagnosis of breast cancer before risk-set entry	Before 1 January 2024 if already due/overdue; otherwise before the date screening becomes due
**Bowel cancer screening**	Diagnosis of bowel cancer before risk-set entry becomes due	Before 1 January 2024 if already due/overdue; otherwise before the date screening
**NHS Health Check**	Any NHS Health Check exclusion condition recorded before risk-set entry, including statin prescription, stroke, hypercholesterolaemia, atrial fibrillation, coronary heart disease, chronic kidney disease, diabetes, heart failure, hypertension, peripheral arterial disease, or palliative care	Before 1 January 2024 if already due/overdue; otherwise before the date the NHS Health Check becomes due
**Adult immunisations**	No service-specific exclusions applied	Not applicable
**Childhood immunisations**	No service-specific exclusions applied	Not applicable

For cancer screening programmes and NHS Health Checks, exclusion criteria will be applied before risk-set entry. Individuals meeting exclusion criteria after entering the risk set will remain eligible and continue contributing follow-up until service completion, death, or administrative

censoring. Exclusions are therefore evaluated at risk-set entry rather than continuously during follow-up.

2.5.2 Secondary outcomes

2.5.2.1 Individual components of CRCI (household-level):

The individual components of the composite outcome (immunisations, cancer screenings, NHS Health Checks) will be analysed separately. This is consistent with international recommendations and with the CRCI framework which highlight the importance of presenting individual components alongside a composite outcome to aid interpretation and transparency[1–4].

- **Definition:** Each will be defined analogously to the primary outcome but restricted to one category of preventive services.
  - immunisations CRCI (rate of completed immunisations per PY of eligibility)
  - Cancer screening CRCI (rate of completed screening per PY of eligibility)
  - NHS Health Checks CRCI (rate of completed checks per PY of eligibility)

2.5.2.2 Healthcare service utilisation:

- **Outcomes:** Annual counts or rates of the following will be estimated per household:
  - GP attendances (all-cause),
  - A & E attendances, (all cause),
  - Emergency hospital admissions (all cause),
  - 30-day hospital re-admission rates (all cause)
- **Exposure:** household person-time (sum of member-year at risk) will be used as the offset.

Statistical methods for analysing these outcomes are described in Section 4

2.5.3 Timepoints of evaluation:

The primary analysis will be conducted after final follow-up, with service completions counted to 30 June 2026.

To address external stakeholder requirements, one interim analysis will be undertaken in April 2026, based on data available up to December 2025. This interim analysis will focus on descriptive summaries and exploratory estimates of intervention effects on primary and secondary outcomes. No formal hypothesis testing will be performed at this stage, and results will be interpreted cautiously. The final analysis, including confirmatory hypothesis testing, will be conducted after trial completion (2026).

The purpose of the interim analysis is to provide early insights into the potential intervention impact in response to political and policy pressure. By design, it is exploratory, with the definitive evaluation reserved for the final analysis.

	**2023**				**2024**				**2025**				**2026**			
**Quarter**	**1**	**2**	**3**	**4**	**1**	**2**	**3**	**4**	**1**	**2**	**3**	**4**	**1**	**2**	**3**	**4**
**Objectives: cluster RCT**																
Steering group formation, trail set up, IRAS application, DAG application, statistical analysis plan			X	X												
Data extract from WSIC								X				X		X		
Statistical analysis, unblinding														X		
Publication preparation, dissemination, application for expanded RCT in further localities																X

**Steering group/PPIE meeting**	X	X	X	X	X	X	X	X	X	X	X	X	X	X	X	X

2.6 Statistical hypotheses

The primary hypothesis will be tested using a two-sided significance level of 5%.

- **Null hypothesis (H0):**

There is no difference in rate of household uptake of preventive services between intervention and control arms (rate CRCI in intervention households is equal to control households).

- **Alternative hypothesis:**

There is a difference in the rate of household uptake of preventive services between trial arms, measured by the CRCI rate per person-year.

Formally:

H0: λ intervention = λ control

H1: λ intervention $\neq$ λ control

Where λ represents the CRCI incidence rate in each trial arm.

Although the intervention is expected to increase uptake, a two-sided test will be used for robustness and consistency with standards RCT practice.

2.7 Sample size

The sample size for this trial is determined primarily by operational and service delivery considerations. Healthcare Central London will fund the recruitment of additional CHWWs for deployment from 2024 in the Lisson Grove, Paddington Green, and Churchill Gardens wards of Westminster, which fall within the most deprived 20% of areas nationally.

In line with the CHWW deployment model, 60 villages, each comprising around 120 households, will be mapped within the eligible areas. These 60 clusters will constitute the full population available for randomisation during the expansion phase and will be allocated in a 1:2 ratio to the intervention and control arms, respectively. This corresponds to approximately 7,200 households in total (2,400 in the intervention arm; 4,800 in the control arm).

The initial sample size estimate registered on clinicaltrials.gov treated the primary outcome as a simple proportion. The trial was designed to provide > 90% power (2α=0.05) to detect an absolute improvement of 10% in the uptake of preventive services assuming a within-period intraclass correlation coefficient (ICC) of 0.15 and baseline uptake of 15% under usual care conditions (demonstrated from pilot evaluation). This calculation assumed a "two-period decay" thus allowing for the within-cluster correlation within the same measurement period to differ to that from a different measurement period. The within-cluster correlation from different periods was assumed to be 80% of the correlation from the same period. For repeated within-household measurements, it was assumed to be a correlation of 0.5. Finally, this calculation assumed that at least 120 households will be included in each village, thus resulting in a total of at least 7,200 households (2,400 randomised to the intervention, and 4,800 randomised to usual care). Under these assumptions, the 60 available clusters will provide at least 90% power to evaluate the intervention's effect on the CRCI, using a two-sided significance level of 0.05. Power calculation was conducted using an online cluster randomised controlled trial calculator (https://clusterrcts.shinyapps.io/rshinyapp/). However, the primary outcome, the Composite Referral Completion Indicator (CRCI), is more accurately evaluated as a rate-based outcome measuring preventive services taken up over varying lengths of follow-up (person-years). Because standard sample size calculators do not natively handle varying person-years, we have updated the power calculation to utilise a more precise and suitable methodology. Accordingly, the updated sample size and statistical power are based on methods appropriate for clustered count/rate outcomes with a logarithmic link and person-years offset, rather than methods designed for simple binary proportions.

The revised power calculations were based on the closed-form formula derived by Li et al. for cluster-randomised trials analysed using Generalized Estimating Equations (GEE)[5]. This comprehensive approach was specifically selected because it directly accommodates several pragmatic features inherent to this trial: the unequal 1:2 allocation ratio, overdispersed count outcomes, random variability in cluster sizes, and unequal lengths of follow-up (person-years) resulting from varying exposure times. The literature demonstrates that ignoring these pragmatic features particularly varying cluster sizes and varying lengths of follow-up can lead to severe underestimation of the required sample size (by up to 27%) and a subsequent loss of statistical power[6]. Since the total number of clusters (N=60) is fixed by deployment, this re-estimation solves for achieved power at the observed sample size rather than calculating a required sample size.

The nuisance parameters (ICC, overdispersion, cluster-size moments, and follow-up moments) were re-estimated from January 2024 to December 2025 interim trial data rather than from the pre-trial historical WSIC data originally planned. This update was made for 2 reasons. First, the interim data represents the actual enrolled households in the 60 trial clusters, making it the most accurate reflection of the study population. Second, the 2022-2023 historical period was closed to the COVID-19 pandemic recovery phase, during which NHS screening and immunisation service uptake was substantially disrupted, risking misleading parameters estimates.

The treatment effect parameter ($\beta 20 = \log(0.25/0.15) \approx 0.511$ was kept fixed at the protocol-specified value throughout and was never re-estimated from interim data. This is critical safeguard against type I error inflation: only the nuisance parameters were updated, not the effect size. This approach is consistent with the nuisance parameter only recalculation framework described by Friede & Schmidli, who demonstrated that this procedure maintains the required power without inflating the type I error rate for overdispersed count outcomes[7]. Harden & Friede further confirmed that nuisance parameter recalculation produces no detectable difference in type I error or power whether conducted with or without knowledge of treatment allocation, provided the treatment effect is no re-estimated[8].

The re-estimation was conducted after unblinding, with arm included as a covariate in the GEE model to obtain an unbiased estimate of overdispersion. This is appropriate at this stage of the trial and is supported by simulation evidence from Grayling & Wason, who found that unblinded nuisance parameter re-estimation performs as well as blinded re-estimation in terms of type I error control, provided the treatment effect estimate itself is not fed back into the recalculation[9].

The re-estimated nuisance parameters are ICC(ρ) = 0.0021, overdispersion(θ)=13.11, mean cluster size(η_m)=73.93 households, and mean household-level person-time(γ_t) = 11.45 person-years. Under these parameters, the trial achieves power > 90% for N =60 clusters to detect an absolute increase of 0.10 CRCI events per person-year (from 0.15 to 0.25), at a two-sided significance level of 0.05. this result is robust across a wide range of sensitivity scenarios: power remains above 90% for ICC values up to ρ=0.05, and above 89% for mean household person-time values down to γ_t=5.0 person-years.

3 Statistical analysis

3.1 Statistical principles

3.1.1 Level of statistical significance

The primary outcome which of the rate of CRCI at the household level will be analysed using a two-sided significance level of 5% (α = 0.05). Point estimates and 95% confidence intervals will be presented for primary and secondary outcomes. All pre-specified sensitivity analyses will use the same two-sided 5% significance level.

3.1.2 Interim analysis

One interim analysis is planned (April 2026) to provide descriptive insights for funders and oversight committees. The interim analysis will summarise the primary and secondary outcomes using descriptive statistics. No hypothesis testing or p-value reporting will be undertaken at the interim stage, as the interim analysis is not intended for inferential decision-making. The trial will continue to full completion regardless of interim findings. A The confirmatory analysis will be done after the final follow-up in mid-2026.

3.1.3 Multiplicity adjustment

Secondary outcomes will be reported using point estimates and 95% confidence intervals. The analysis of secondary outcomes will focus on estimation rather than significance testing. Accordingly, no adjustment for multiplicity will be applied, and p-values will not be interpreted as confirmatory evidence of effect but will be reported where relevant for completeness. Any exploratory p-values that may appear in supplementary output will not be interpreted as inferential evidence.

3.1.4 Statistical software

All analyses will be performed using R (version 4.3.1 or above).

3.2 Datasets analysed

3.2.1 Intention-to-treat (ITT) population

The ITT population will consist of all eligible households in villages randomised to either intervention or control, regardless of whether the allocated intervention was delivered. Analyses of the primary outcome (CRCI) will be conducted on the ITT population.
Households with no eligible preventive services during a given trial year will be excluded from the denominator in the primary analysis but will be described separately.

3.2.2 Per-protocol (PP) population

The PP analysis will be conducted at the household level, conditional on village-level implementation in the intervention arm (CHWW in post during the trial period). Within the implemented intervention villages, PP households are those on the CHWW assignment list with the documented CHWW visit during the trial period.

3.3 Subject disposition

Analyses will follow cluster-randomised CONSORT guidelines, and the flow of villages, households, and individuals through the trial will be displayed in a CONSORT diagram.

3.4 Handling of protocol deviations

Protocol deviations (e.g., households reallocated due to boundary changes) will be recorded and reported. The number and proportion of households affected will be described.

4 Planned analyses

4.1 Baseline characteristics and comparisons

4.1.1 Cluster (village) characteristics

Cluster-level characteristics (e.g., geographic region, deprivation index, number of households per village) will be summarised by randomised arm as outlined in Appendix 1 (table 1).

4.1.2 Household characteristics

Household-level baseline characteristics (e.g., household size, composition) will be described by intervention group.

4.1.3 Individual characteristics

Individual characteristics of household members (e.g., age distribution, sex, ethnicity) will be summarised descriptively by trial arm.
Discrete variables will be presented as counts and percentages. Percentages will be calculated according to the number of clusters or individuals with available data. Continuous variables will be summarised using mean and standard deviations (SD), and median and interquartile range (Q1-Q3).

4.1.4 Presentation and interpretation

Baseline summaries will be presented in tabular form (Appendix tables).
No formal statistical tests of baseline will be performed, in line with CONSORT recommendations for cluster randomised trials.
These summaries will provide descriptive context to characterise the study population and confirm comparability between trial arms.

4.2 Analysis of the primary outcome

4.2.1 Main analysis

The primary outcome, CRCI, is defined as the rate of completion of preventive services opportunities per person-year at risk over the study period.
The primary outcome will be analysed at the household level using a mixed-effects GLMM with a log link and a log(person-time) offset.

- **Response:** number of completed service events for a household.
- **Offset:** log of total person-years at risk for that household.
- **Fixed effect:** treatment arm (intervention vs control) and randomisation stratum (North vs South).
- **Random effects:** random intercepts for villages (clusters) to account for the cluster design (within-cluster correlation).
- **Effect measure:** the intervention effect will be expressed as rate ratio (RR) for service completion with 95% confidence intervals. For interpretability, marginal absolute rate differences (i.e. the mean difference in completed preventive service events per 100 person-years at risk between arms) will be reported. These marginal predictions will be obtained from the fitted regression model, averaged over the study population's covariate distribution.

4.2.2 Overdispersion

The Poisson model assumes that the variance of counts equals the mean. If overdispersion is detected (i.e. variance substantially exceeding the mean) the analysis will instead use a mixed-effects negative binomial regression, which includes an additional dispersion parameter.

4.2.3 Adjusted analyses

The primary model will be extended to include additional baseline covariate to improve precision while preserving the randomisation estimated:

- Household size (continuous or categorical).

Adjusted effects will be reported as rate ratios and marginal risk differences with 95% CIs. No variables that could be influenced by the intervention (e.g., CHWWs visits) will be included in the adjusted model.

4.2.4 Assumption checks and convergence

Overdispersion: assessed using Pearson residual deviance and likelihood ratio tests.
Model convergence: if the mixed-effects model fails to converge, a Generalised Estimating Equation (GEE) with exchangeable correlation structure and robust sandwich standard errors will be fitted as an alternative.

Exploratory methodological analysis (Absolute effects):

While the primary GLMM evaluates the intervention's relative effect (Incidence Rate Ratio), the CONSORT statement explicitly recommends reporting both relative and absolute effects (such as the risk or rate difference) to fully capture an intervention's public health impact. Furthermore, following ICH-E9(R1) guidelines, the causal estimand must be strictly determined by the study's primary research goal rather than the default outputs of standard statistical software. Because the overarching goal of this pragmatic trial is to evaluate the real-world, population-level impact of the CHWW model as it is rolled out across villages, estimating the effect on the absolute scale is essential[1].

However, estimating adjusted absolute differences in cluster-randomised trials presents specific methodological challenges. Recent simulation studies demonstrate that extracting absolute differences using standard individual-level modeling approaches is highly problematic; GLMMs frequently underestimate standard errors when estimating absolute effects, while Generalized Estimating Equations (GEE) and subsequent G-computation suffer from severe model convergence failures (failing to converge in up to 25% of complex scenarios)[10].

Therefore, to overcome these limitations and accurately target the cluster-level effect, we will conduct a pre-specified methodological sub-study utilising Cluster-Level (Hierarchical) Targeted Maximum Likelihood Estimation (TMLE) to estimate the covariate-adjusted marginal absolute difference in the CRCI. Recent methodological evidence proves that hierarchical TMLE provides the most robust performance for estimating absolute differences in cluster trials, successfully avoiding the bias, standard error inaccuracies, and severe convergence issues that plague standard models. Crucially, while the primary GLMM analysis will adjust for a mix of individual- and cluster-level variables, the hierarchical TMLE will be adjusted exclusively for individual-level covariates (household size, age, and sex). Recent literature demonstrates this yields strictly superior performance specifically optimizing Type I error and coverage rates when calculating absolute differences using cluster-level TMLEs[10].

Finally, to properly account for the complex data structure, including varying lengths of follow-up (person-time) and right-censoring, this sub-study will follow the recent best-practice guidelines established for the application of TMLE to time-to-event outcomes[11]. In accordance with these methodological guidelines, the trial's follow-up period will be discretized into appropriate sub-periods. This allows the TMLE algorithm to natively handle varying person-time and missing-at-random censoring by recursively estimating the counterfactual absolute event probabilities over time, thereby generating robust absolute rate reductions without relying on the restrictive parametric assumptions of a standard regression offset.

4.2.5 Handling of household composition changes

Household composition may change during the trial due to births, deaths, or individuals moving in or out. For the purposes of primary analysis, household membership will be defined at and treated as fixed throughout follow-up.

Preventive service eligibility and uptake will therefore be based on the set of household members present at 1st January 2024.

This pragmatic approach reflects the design of the intervention and ensure feasibility when working with routinely collected data. Adjusting numerator and denominators for partial residency (e.g., scaling service eligibility according to time spent in the household) would introduce additional complexity and is unlikely to materially affect estimates or statistical power[4]. However, we will allow for variable observation time: individuals contribute person-time and outcomes only while registered and under observation (until death, or study end). Household denominators will therefore reflect the aggregated observation time of study cohort , consistent with at-risk approach planned for this trial.

4.2.6 Handling missing data

Preventive service completions are ascertained from WSIC routine data feeds. We will assume complete capture for NHS services within WSIC: if a completion is not recorded during follow-up window, it will be treated as not completed. Under this assumption, outcome imputation is not planned.

4.2.7 Sensitivity analyses
Sensitivity analyses will be conducted to assess the robustness of the estimated intervention effect. Unless specified otherwise, results will be presented as rate ratios with 95% CIs, alongside absolute rate differences per 100 person-years.

1. COVID-19 Recovery Baseline Adjustment

The primary analysis incorporates a continuous 24-month pre-trial baseline CRCI covariate adjustment (1 January 2022 – 31 December 2023) to capture seasonality and provide statistical stability. However, data from 2022 may reflect artificially low preventive service uptake due to the ongoing recovery from the COVID-19 pandemic. While such lingering effects are expected to be balanced across both study arms by randomisation (and thus unlikely to introduce bias), a highly distorted 2022 baseline may exhibit reduced correlation with the trial's prospective outcome data. This reduced correlation would make the baseline covariate less effective at explaining variance, potentially reducing the statistical power and efficiency of the primary GLMM.

Therefore, a sensitivity analysis will be conducted by replacing the 24-month baseline covariate with a 12-month window (1 January 2023 – 31 December 2023). This analysis will evaluate the impact of this potential pandemic-related distortion on model efficiency and confirm the robustness of the primary treatment effect.

2. Per-protocol analysis

Because the primary analysis follows an intention-to-treat (ITT) principle, it evaluates the offer of the CHWW intervention regardless of actual uptake. To evaluate the efficacy of the intervention when actually delivered, a per-protocol analysis will be conducted. The primary GLMM will be re-run by restricting the intervention arm cohort exclusively to households that actively received the CHWW intervention (defined as having documented evidence of receiving at least one CHWW visit during the trial follow-up period). This compliant intervention cohort will be compared against the full control group.

Each sensitivity analysis will report the intervention effect estimate and 95% CIs alongside the primary model results. A summary table and forest plot will display all sensitivity estimates to facilitate comparison.

4.3 Analysis of secondary outcomes
All secondary outcomes will be analysed in both the ITT and PP populations as defined in section 3.2. Results will be presented as effect estimates with 95% CIs. These analyses are exploratory and hypothesis-generating, results will be interpreted with caution and without formal multiplicity correction.

4.3.1 Components of CRCI
Each component of the CRCI will be analysed separately at the household level:
- Immunisation CRCI.
- Cancer screening CRCI.
- NHS Health Checks.

Analysis approach
- **Definition:** each component CRCI is the rate of completed opportunities per person-year at risk for that service, aggregated at the household level during the study period.
- **Model:** mixed-effects GLMM with log(person-time) offset, random intercept for village, and fixed effects for intervention arm and randomisation stratum.
- **Estimates reported:** rate ratios (RRs) with 95% CIs.

These analyses are exploratory; no adjustment for multiplicity will be applied.

4.3.2 Healthcare service utilisation
Healthcare utilisation outcomes include:
- GP consultations (all-cause),
- A & E attendances, (all cause),

- Emergency hospital admissions (all cause),
- 30-day re-admission rates (all cause)

Analysis approach:

- Outcomes will be assessed as counts at the household-level.
- **Exposure:** household person-time (registration-adjusted day) will be used as the offset in rate models.
- **Main model:** mixed-effects GLMM with:
  - **Fixed effects:** intervention arm and randomisation stratum.
  - **Random effects:** village intercepts.
  - **Offset:** log(person-time).

Effect measure

- Effects will be reported as adjusted RRs with 95% CIs.

4.3.3 Interpretation

Analyses of CRCI components will help identify which services are most responsive to the CHWW intervention, complementing the composite CRCI. Analyses of healthcare utilisation will explore whether the intervention is associated with shifts in service use patterns, such as reduced emergency care.

All secondary outcomes are exploratory and hypothesis-generating, results will be interpreted with caution and without formal multiplicity correction.

5 References

1. Abraham J. International Conference On Harmonisation Of Technical Requirements For Registration Of Pharmaceuticals For Human Use. In: Tietje C, Brouder A, editors. Handbook of Transnational Economic Governance Regimes [Internet]. Brill | Nijhoff; 2010 [cited 2026 Jun 2]. p. 1041–53. Available from: https://brill.com/view/book/edcoll/9789004181564/Bej.9789004163300.i-1081_085.xml doi:10.1163/ej.9789004163300.i-1081.897

2. Cordoba G, Schwartz L, Woloshin S, Bae H, Gotzsche PC. Definition, reporting, and interpretation of composite outcomes in clinical trials: systematic review. BMJ. 2010 Aug 18;341(aug18 3):c3920–c3920. doi:10.1136/bmj.c3920

3. Junghans C, Antonacci G, Williams A, Harris M. Learning from the universal, proactive outreach of the Brazilian Community Health Worker model: impact of a Community Health and Wellbeing Worker initiative on vaccination, cancer screening and NHS health check uptake in a deprived community in the UK. BMC Health Serv Res. 2023 Oct 12;23(1):1092. doi:10.1186/s12913-023-10084-8

4. Watt H, Harris M, Noyes J, Whitaker R, Hoare Z, Edwards RT, et al. Development of a composite outcome score for a complex intervention - measuring the impact of Community Health Workers. Trials. 2015 Dec;16(1):107. doi:10.1186/s13063-015-0625-1

5. Li D, Zhang S, Cao J. Incorporating pragmatic features into power analysis for cluster randomized trials with a count outcome. Stat Med. 2020 Nov 30;39(27):4037–50. doi:10.1002/sim.8707

6. Zhan D, Xu L, Ouyang Y, Sawatzky R, Wong H. Methods for dealing with unequal cluster sizes in cluster randomized trials: A scoping review. Sarkis-Onofre R, editor. PLOS ONE. 2021 Jul 29;16(7):e0255389. doi:10.1371/journal.pone.0255389

7. Friede T, Schmidli H. Blinded sample size reestimation with count data: Methods and applications in multiple sclerosis. Stat Med. 2010 May 10;29(10):1145–56. doi:10.1002/sim.3861

8. Harden M, Friede T. Sample size recalculation in multicenter randomized controlled clinical trials based on noncomparative data. Biom J. 2020 Sep;62(5):1284–99. doi:10.1002/bimj.201900138

9. Grayling M, Mander A, Wason J. Blinded and unblinded sample size re-estimation procedures for stepped-wedge cluster randomized trials [Internet]. arXiv; 2017 [cited 2026 Sep 1]. Available from: http://arxiv.org/abs/1710.02683 doi:10.48550/arXiv.1710.02683

10. Pereira Macedo JA, Giraudeau B, Escient Collaborators. Estimating an adjusted risk difference in a cluster randomized trial with individual-level analyses. Stat Methods Med Res. 2025 Jan;34(1):26–44. doi:10.1177/09622802241293783

11. Talbot D, Diop A, Mésidor M, Chiu Y, Sirois C, Spieker AJ, et al. Guidelines and Best Practices for the Use of Targeted Maximum Likelihood and Machine Learning When Estimating Causal Effects of Exposures on Time-To-Event Outcomes. Stat Med. 2025 Mar 15;44(6):e70034. doi:10.1002/sim.70034

6 Appendix

6.1 Proposed outputs

Table 1: Trial population by allocation group

Characteristics	Intervention	Control	Total
Number of villages			
Number of households			
Number of individuals			
Mean household size (SD)			
Median (IQR) household size			
Proportion of female household heads (%)			
Median household income band			
Total follow-up person-years			
Note: This table summarises the baseline population characteristics at household and village levels.			

Table 2: Baseline characteristics of households

Characteristics	Intervention	Control	Total
Age (years)			
Mean (SD)			
Median (IQR)			
Sex			
Male	XX (XX.X %)	XX (XX.X %)	XX (XX.X %)
Female	XX (XX.X %)	XX (XX.X %)	XX (XX.X %)
Ethnicity			
White			

**Asian or Asian British**			
**Black or Black British**			
**Mixed**			
**Other ethnic group**			

Table 3: baseline medical history

Table 3: preventive health care service eligibility at household level

**Service**	**Eligible Households**		
	**Intervention (N=)**	**Control (N=)**	**Total (N=)**
**Immunisations**			
**Influenza**			
**MMR (1st dose)**			
**MMR (2nd dose)**			
**DTaP/ IPV/Hib/ HepB (1st dose)**			
**DTaP/ IPV/Hib/ HepB (2nd dose)**			
**DTaP/ IPV/Hib/ HepB (3rd dose)**			
**Rotavirus (1st dose)**			
**Rotavirus (2nd dose)**			
**Meningitis B MenB (1st dose)**			
**Meningitis B (MenB) (2nd dose)**			
**Meningitis B (MenB) (3rd dose)**			
**Pneumococcal Polysaccharide Vaccine (PPV)**			

**HiB/ Meningitis C (MenC)**			
**DTaP/IPV**			
**Td/IPV**			
**MenACWY**			
**Human Papillomavirus Vaccine (HPV)**			
**COVID-19 Vaccine**			
**RSV vaccine**			
**Shingles**			
**Cancer screening**			
**Bowel cancer**			
**Breast cancer**			
**Cervical cancer**			
**NHS Health Checks**			

Table 4: health service use and hospital admission

**Variable**	**Intervention (N=)**	**Control (N=)**	**Total (N=)**
**GP visits (all-cause)**			
**Mean (SD)**			
**Median (Q1;Q3)**			
**Min Max**			
**Accidents & Emergency attendances (all-cause)**			

**Mean (SD)**			
**Median (Q1;Q3)**			
**Min Max**			
**Emergency hospital admission (all-cause)**			
**Mean (SD)**			
**Median (Q1;Q3)**			
**Min Max**			
**30-day hospital readmission rates (all-cause)**			
**Mean (SD)**			
**Median (Q1;Q3)**			
**Min Max**			

Table 5: preventive healthcare services and healthcare utilisation outcomes – descriptive analysis

**Variable**	**Intervention (N=)**	**Control (N=)**	**Total (N=)**
**Composite outcome**			
**Mean CRCI (SD)**	**XXX.X (XXX.X)**	**XXXX**	
**Median CRCI (IQR)**	**XXXX**	**XXXX**	
**Min max**			
**Individual components of CRCI**			
**Immunisation CRCI**			
**Cancer screening CRCI**			
**NHS Health Checks**			
**Healthcare utilisation**			
**GP attendances**			

**A&E attendances**			
**Emergency hospital admission**			
**30-day readmission rate**			

Table 6: Primary outcomes – model results

**Outcome :**	**Unadjusted model**			**Adjusted model**		
	**N**	**Estimate (95% CI)**	**P-value**	**N**	**Estimate (95% CI)**	**P-value**
**composite CRCI (rate per person-year)**	**XXXX**	**XX.XX(XX.XX to XX.XX)**	**0.XXXX**	**XXXX**	**XX.XX(XX.XX to XX.XX)**	**0.XXXX**
**Absolute difference (per 100 PY)**		**XXXX**			**XXXX**	

Table 7: secondary outcomes – model results

**Outcome :**	**Unadjusted model**			**Adjusted model**		
	**N**	**Estimate (95% CI)**	**P-value**	**N**	**Estimate (95% CI)**	**P-value**
**CRCI component outcomes**						
**Immunisation**						
**Cancer screening**						
**NHS Health Checks**						
**Healthcare utilisation outcomes**						
**GP consultations**						
**A & E attendances**						
**Emergency hospital admissions**						
**30-day readmission rate**						

Table 8 protocol violations and deviations

**Cause**	**Intervention (N=)**	**Control (N=.)**

**Any protocol violation or deviation**	**nnn xx%**	**Nnn xx%**
**Any protocol violation**		
**Category 1**		
**Category 2**		
**Any protocol deviation**		
**Category 1**		
**Category 2**		

Table 9: sensitivity analyses of the primary outcome

**Model**	**Unadjusted**			**Adjusted**		
	**N**	**RR (95%CI)**	**P-value**	**N**	**RR (95%CI)**	**P-value**

Table 10: number and proportion of households with different household size for preventive healthcare services in the intervention and control groups.

**Household size**	**Intervention**	**Control**	**Total**
**Immunisations**			
**1**			
**2**			
**3**			
**4**			
**5 or more**			
**Cancer screening**			
**1**			
**2**			
**3**			
**4**			
**5 or more**			
**NHS Health Checks**			
**1**			
**2**			
**3**			
**4**			
**5 or more**			
