# Supplementary material for: Community health and well-being workers (CHWWs) for preventive healthcare services uptake in the UK: a cluster-randomised controlled trial protocol
Source: BMJ Open. 2026 Oct 23;16(10):e124582. doi: 10.1136/bmjopen-2026-124582 (PMC13711556; doi:10.1136/bmjopen-2026-124582)
Supplement: Supplementary file 3 [file bmjopen-2026-124582.reviewer_comments.pdf]

PEER REVIEW HISTORY

BMJ Open publishes all reviews undertaken for accepted manuscripts. Reviewers are asked to complete a checklist review form and are provided with free text boxes to elaborate on their assessment. These free text comments are reproduced below.

ARTICLE DETAILS

Title (Provisional)

Community Health and Wellbeing Workers (CHWWs) for Preventive Healthcare Services Uptake in the UK: a cluster-randomised controlled trial protocol

Authors

Gadi, Rawan; Junghans Minton, Cornelia; Alkhatib, Ahmad; Woodcock, Thomas; Peiris, David; Berwanger, Otavio; Billot, Laurent; Harris, Matthew

---

VERSION 1 - REVIEW

**Reviewer**	**1**
**Name**	**Napierala, Hendrik**
**Affiliation**	**Charité – Universitätsmedizin Berlin, Institute of General Practice and Family Medicine**
**Date**	**10-Aug-2026**
**COI**	**None**

The manuscript reports a protocol for a cluster RCT on the effect of CHWWs on the uptake of preventive services. The trial is of high importance to a wider readership.

The manuscript is well written, but I believe that some additional information should be added to the main text (see below).

I have the following comments:

- Eligbility criteria on cluster level should be justified (especially: “villages”). I could not grasp why you defined it like that.

- Intervention arm could be supplemented by a table using the TIDIER framework.

- How long is the training for CHWWs? Is there no prerequisite to be eligible as a CHWW?

- Some of the important aspects (i.e. concerning confidentiality, further statistical analyses) in the SAP should be added to the manuscript itself.

- Study limitations could be reported in more detail than just the article summary.

- SPIRIT checklist:

a) information should be in the manuscript (i.e. Version date and number, item 2)

b) 5: is there no further underlying protocol that was acknowledged by the ethics committee?

c) Missing: 15d

- Conflicts of interest need to be added to the protocol

VERSION 1 - AUTHOR RESPONSE

REVIEWER1 COMMENTS

Comment 1: *village/cluster eligibility justification "Eligibility criteria on cluster level should be justified (especially: "villages"). I could not grasp why you defined it like that.*

Response:

The term "village" refers to the pre-established operational units of the CHWW programme , not an arbitrary label invented for this trial. We have added a clarifying sentence to the cluster eligibility criteria section. The revised text now reads:

"Eligible clusters are geographically defined areas referred to as 'villages' within the CHWW programme - the pre-established operational units of intervention delivery - each comprising approximately 120 households located within the targeted LSOAs and covered by participating primary care networks. Villages represent the natural and necessary unit of clustering for this trial. Each CHWW is assigned to a single village and operates within it universally, proactively engaging all households."

Comment 2: *TIDieR table "Intervention arm could be supplemented by a table using the TIDIER framework."*

Response:

We have added a full TIDieR (Template for Intervention Description and Replication) checklist table as Supplementary Appendix 3. A reference to this table has been added at the end of the Interventions section in the main manuscript:

"Full intervention description in accordance with the TIDieR checklist is provided in supplementary material 2."

Comment 3: CHWW training and prerequisites

"How long is the training for CHWWs? Is there no prerequisite to be eligible as a CHWW?"

Response:

We have expanded the Training section to include details on CHWW prerequisites, training duration, content, and ongoing support. The revised text now reads:

"All CHWWs complete a standard training programme specified by the commissioning organisations prior to deployment. CHWWs are recruited based on their motivation for the role, residency in or close to the local neighborhood, emotional intelligence, and commitment to helping others. No prior professional qualification is required beyond secondary school education. Following recruitment, CHWWs complete three to four weeks of training covering online resources, Just Listening, data management, confidentiality, lone working, safeguarding, and common health conditions. On-the-job support is provided through weekly clinical

supervision sessions with a lead GP, discussion of complex cases, and identification of learning opportunities. Reflexive practice is supported through case write-ups and vignettes reviewed with line managers. Note: a CHWW Level 3 certificate and apprenticeship has since become available through the Royal Society of Public Health to support scaling of the role nationally, but this was not a requirement during the intervention period of this study. “

Comment 4: SAP content: confidentiality and statistical analyses
“Some of the important aspects (i.e. concerning confidentiality, further statistical analyses) in the SAP should be added to the manuscript itself.”

Response:
We have substantially restructured and expanded the Statistical Methods section, incorporating the following details from the SAP: explicit statement of the intention-to-treat (ITT) principle; randomisation stratum (North vs South) as a fixed effect; overdispersion check with negative binomial regression as a fallback; GEE with robust sandwich standard errors as a convergence fallback; household composition handling (fixed at 1 January 2024). The confidentiality and data management section were already present in the manuscript and remains unchanged. Full statistical analysis details are provided in the SAP (supplementary file 3).

Comment 5: study limitation *“Study limitations could be reported in more detail than just the article summary.”*
Response:
We have revised and expanded the Strengths and Limitations box. In line with BMJ Open guidelines, this section is limited to five bullet points relating to methodology. We have added a new bullet addressing the geographical scope of the study and its implications for generalisability:
“The study is conducted within the London Borough of Westminster, targeting the most socioeconomically deprived LSOAs; while this focus strengthens internal validity and relevance to deprived urban communities, findings may not be directly generalisable to CHWW programmes operating in different geographic, demographic, or healthcare system contexts.”

Comment 6: protocol version and date *“SPIRIT checklist: information should be in the manuscript (i.e. Version date and number, item 2)”*
Response:
We have added the protocol version and date to the opening paragraph of the Methods section. The text now reads:
“This protocol (Version 2, June 2026) has been prepared in accordance with the SPIRIT (Standard Protocol Items: Recommendations for Interventional Trials) reporting guidelines.”
The SPIRIT checklist item 2 has been updated accordingly.

Comment 7: Ethics committee protocol “*is there no further underlying protocol that was acknowledged by the ethics committee?”*
Response:
Yes, the study was reviewed and approved by the Health Research Authority (HRA reference: 23/HRA/4972, 20th December 2023). Additionally, the use of routinely collected WSIC data falls under an existing global ethics agreement (REC reference:

REC18/WM/0323/IRAS252449). Both references are stated in the Ethics and Dissemination section of the manuscript.

Comment 8: SPIRIT Item 15d: *concomitant care "SPIRIT checklist: Missing: 15d."*
Response:
We have updated SPIRIT checklist item 15d. Concomitant care is addressed in the manuscript: "no concomitant care is prohibited in either arm" and the relevant page reference has been added to the checklist.

Comment 9: conflict of interest *"Conflicts of interest need to be added to the protocol"*
Response:
We have added a competing interest's statement to the Declarations section confirming that all authors declare no competing interests.

Additional update
Following the submission of the original manuscript, we conducted a pre-specified power re-estimation using observed interim cohort data, this update represents the actual enrolled households in the 60 trial clusters, making it the most accurate reflection of the study population. The updated power calculation, using the Li et al. (2020) GEE closed-form formula for cluster-RCT power with count/rate outcomes, yielded > 90% achieved power (ICC $\rho = 0.0021$; mean person-time $\gamma_t = 11.45$ person-years). This reflects the fixed number of clusters determined by the actual programme deployment rather than a target power calculation. The sample size section of the manuscript has been updated accordingly. Full details are provided in the updated SAP (Supplementary Appendix 3).
